# Characteristics of Urinary Tract Infections in Patients with Diabetes from Timișoara, Romania: Prevalence, Etiology, and Antimicrobial Resistance of Uropathogens

**DOI:** 10.3390/medicina60111870

**Published:** 2024-11-14

**Authors:** Teodora Sorescu, Monica Licker, Romulus Timar, Corina Musuroi, Delia Muntean, Adela Voinescu, Dan Dumitru Vulcanescu, Andrei Cosnita, Silvia-Ioana Musuroi, Bogdan Timar

**Affiliations:** 1 Department of Internal Medicine II: Diabetes, Nutrition, Metabolic Diseases, and Systemic Rheumatology, “Victor Babes” University of Medicine and Pharmacy, 300041 Timisoara, Romania; sorescu.teodora@umft.ro (T.S.); timar.romulus@umft.ro (R.T.); bogdan.timar@umft.ro (B.T.); 2 Department of Diabetes, Nutrition and Metabolic Diseases, “Pius Brînzeu” Emergency Clinical County Hospital, 300723 Timisoara, Romania; 3 Microbiology Department, Multidisciplinary Research Center of Antimicrobial Resistance, “Victor Babes” University of Medicine and Pharmacy, 300041 Timisoara, Romania; corina.musuroi@umft.ro (C.M.); muntean.delia@umft.ro (D.M.); adela.voinescu@umft.ro (A.V.); dan.vulcanescu@umft.ro (D.D.V.); 4 Microbiology Laboratory, “Pius Brinzeu” Emergency Clinical County Hospital, 300723 Timisoara, Romania; 5 Doctoral School, “Victor Babes” University of Medicine and Pharmacy, 300041 Timisoara, Romania; silvia.musuroi@umft.ro; 6 Department IX, Surg & Ophthalmol, “Victor Babes” University of Medicine and Pharmacy, 300041 Timisoara, Romania; cosnita.dan@umft.ro; 7 Department of Clinical Practical Skills, “Victor Babes” University of Medicine and Pharmacy, 300041 Timisoara, Romania

**Keywords:** urinary tract infections, diabetes, uropathogens, antimicrobial resistance, multidrug-resistant bacteria

## Abstract

*Background and Objectives*: Diabetic patients are more likely to develop infections compared to the general population, especially urinary tract infections (UTIs). The aim of this study was to assess the prevalence of UTIs in a population of patients with diabetes (DM) from Romania, to identify the most common uropathogens and their antimicrobial resistance (AMR) patterns, as well as to determine the correlations between resistance behavior and particularities of patients with UTIs according to DM type. *Materials and Methods*: The hospital records of 1282 type 1 (T1D) and type 2 DM (T2D) adult inpatients who were ordered urine cultures during hospitalization were reviewed, and all 241 patients who presented a positive urine culture were included in the present study analysis. *Results*: The prevalence of UTIs in diabetic patients was 18.8% and higher in patients with T2D vs. T1D. Patients with UTIs and T2D had a significantly older age, longer duration of DM, higher waist circumference and body mass index, lower levels of estimated glomerular filtration rate, and more frequent chronic complications of DM than patients with T1D. *E. coli* was the most frequently isolated uropathogen (56.4%), with a significantly higher incidence for T2D, followed by *K. pneumoniae* (12.9%) and *Enterococcus* spp. (9.5%). Although the acquired resistance phenotypes were more frequently isolated in T2D patients (over 90% of the multidrug-resistant and extended-spectrum beta-lactamase-producing isolates, respectively, and 75% of the total carbapenem-resistant organisms), no statistically significant correlation was found regarding the distribution of AMR patterns in the two types of DM. *Conclusions*: The present study brings new data regarding the prevalence of UTIs in diabetic patients from Western Romania. By identifying the spectrum of uropathogens and their AMR pattern, this paper may contribute to improving UTI management in diabetic patients, thus reducing antibiotic overuse and preventing recurrent UTIs.

## 1. Introduction

Diabetes mellitus (DM) is a serious global health problem affecting 537 million adults worldwide, and this number is set to escalate to 783 million by 2045, according to International Diabetes Federation (IDF) projections [1]. In Romania, IDF data from 2021 indicate a DM prevalence of 8.4% [1]. Over 90% of people with DM have type 2 DM (T2D) [1], a metabolic disorder primarily marked by insulin resistance and relative insulin deficiency, often associated with obesity, genetic predisposition, and environmental factors [2]. Type 1 DM (T1D) is an autoimmune condition typically affecting children and young adults, characterized by the destruction of pancreatic beta cells, resulting in absolute insulin deficiency and the need for lifelong insulin therapy [2]. Persistently high blood glucose levels are associated with increased morbidity and mortality among people living with DM [3], placing a substantial burden on healthcare systems [4] with worldwide health expenditures of at least 966 billion dollars in 2021 [2].

Diabetic patients are more likely to develop infections compared to people without DM [5,6,7,8], urinary tract infections (UTIs) being among the most common of them [9]. The increased prevalence of UTIs in diabetic patients may result from several host-related mechanisms: impairments in the immune system, incomplete bladder emptying due to autonomic neuropathy, the presence of glycosuria due to poor metabolic control of DM, and increased adherence in bacterial strains to the uroepithelial cells [9,10,11].

The clinical presentation of diabetes-associated UTIs varies from patients with asymptomatic bacteriuria (ASB) to cystitis or pyelonephritis, which can more often lead to serious complications and potentially life-threatening conditions, such as emphysematous cystitis and emphysematous pyelonephritis, renal and perinephric abscesses, urosepsis and bacteremia [9,12,13]. The most common uropathogens involved in UTIs in diabetic patients were found to be *Escherichia coli* and other *Enterobacterales* [10,11,12], similar to those isolated from non-diabetic people [14], but unusual or antibiotic-resistant pathogens and fungal infections occur more frequently among patients with DM [12]. Therefore, UTIs in diabetic patients should be considered as complicated UTIs [10], requiring a prompt diagnosis and early treatment [14].

Few studies have been conducted in recent years regarding the prevalence of UTIs in Romania, which ranges between 17% and 25% [15,16]. However, there is even less evidence concerning these infections in the diabetic population [17]. Furthermore, the vast majority of studies in the literature have evaluated the characteristics of UTIs in patients with DM in general, and in those with T2D in particular, and only a few have been conducted in patients with T1D [18]. Therefore, we have undertaken the present study to assess the prevalence of UTIs in a population of diabetic patients from Timișoara, Romania, and to identify the most common uropathogens and their antimicrobial resistance (AMR) pattern, as well as the correlations between resistance behavior and characteristics of patients with UTIs according to DM type.

## 2. Materials and Methods

### 2.1. Study Design, Setting and Population

A hospital-based, retrospective, observational study was carried out from January 2023 until December 2023 in the Diabetes, Nutrition and Metabolic Diseases (DNMD) Clinic from the ‘Pius Brînzeu’ Emergency Clinical County Hospital in Timișoara, Romania. This institution is a tertiary-care university-affiliated teaching hospital, providing healthcare services for the Western region of Romania, with a capacity of 1174 beds, among which 40 serve the DNMD Clinic.

Patients admitted to the DNMB Clinic were enrolled in the study if the following criteria were present: patients with the diagnosis of T1D or T2D, aged 18 years and older, who were ordered urine cultures during hospitalization. Exclusion criteria were represented by patients with a type of DM other than T1D or T2D (like gestational DM), those who did not have urine samples collected for urine cultures, or those who have been taking antimicrobial drugs for the last two weeks prior to the admission. Duplicate samples from the same patient, for which the same pathogen was identified, were disregarded.

The study was approved by the Hospital Ethical Committee (approval no. 487/25/09/2024).

### 2.2. Study Protocol

Hospital records of all the enrolled patients were reviewed. The collected data included demographics, anthropometric characteristics (body mass index—BMI and waist circumference), information about DM (type, duration, and glycemic control, evaluated by the glycated hemoglobin—HbA1c), presence of acute (diabetic ketoacidosis—DKA) and chronic complications of DM (retinopathy, chronic kidney disease—CKD, coronary artery disease, cerebrovascular disease, peripheral artery disease, neuropathy), and comorbidities commonly associated with DM (hypertension and dyslipidemia). Data regarding UTI diagnoses, etiology, antimicrobial susceptibility testing (AST), and renal function (assessed using serum creatinine and estimated glomerular filtration rate—eGFR—which was calculated during hospitalization using CKD-EPI creatinine equation 2021) were also collected. The decision to order a urine culture and the diagnosis of UTIs was established by the attending physician during hospitalization.

### 2.3. Identification of Isolated Microorganisms and AST

The Microbiology Laboratory protocols were followed for the identification of pathogens and the AST. The Matrix Assisted Laser Desorption/Ionization Time-of-Flight Mass Spectrometry (MALDI Biotyper, Bruker, Bremen, Germany) and the VITEK^®^ 2 Compact (BioMerieux, Marcy l’Etoile, France) equipment were used for the identification of pathogens, and the last one was also used to perform AST with minimum inhibitory concentration (MIC) determination. The AST was interpreted in accordance with the EUCAST Clinical Breakpoint Tables v. 13.0.

Classifications based on acquired resistance phenotypes were applied to clinically significant bacteria:

– Methicillin-resistant *S.* *aureus* (MRSA) were identified as isolates with a minimum inhibitory concentration of (MIC) > 2 mg/L to oxacillin;
– Extended-spectrum beta-lactamases (ESBLs) producing Gram-negative bacilli (GNB) were defined as resistant to all penicillins/cephalosporins;
– Carbapenem-resistant organisms (CROs) were defined as *Enterobacterales* and non-fermentative GNB with MIC ≥ 4 mg/L to imipenem and ≥ 8 mg/L to meropenem;
– Multidrug-resistant bacteria (MDR) were defined as possessing acquired resistance mechanisms to at least one antibiotic from three or more classes of active antibiotics for a given species;
– Extensively drug-resistant bacteria (XDR) were defined as resistant to at least one agent from all antimicrobial classes except one or two classes.

### 2.4. Statistical Analysis

Data were collected and analyzed using MedCalc^®^ Statistical Software version 20.211 (MedCalc Software Ltd., Ostend, Belgium, 2023) and are presented as medians and interquartile ranges for continuous variables, and as the number of cases and percent from total for categorical variables. The Mann–Whitney U test was used to assess the significance of the differences between groups when testing continuous variables. A chi-square analysis was used to test categorical variables. The odds ratio (OR) was calculated to determine the risk of occurrence of AMR patterns after exposure or non-exposure to a certain factor. A logistic regression model was used to analyze the risk factors in cases in which the exposure was quantified through continuous variables. The selection of predictors in the model followed a stepwise consecutive–prospective acceptance process (predictors being accepted at a significance level of *p* ≤ 0.1), the chosen model being the one that best explained the emergence of AMR patterns. To evaluate the diagnostic performance of eGFR as a predictor for the presence of MDR and ESBL isolates, respectively, we conducted the receiver-operating characteristics (ROC) curve analysis. The predictive performance was measured using sensitivity and specificity. The area under the ROC curve (AUC) was calculated to quantify the overall accuracy of eGFR as a predictive marker for MDR and ESBL isolates; an AUC of 1 indicated perfect discrimination, while an AUC of 0.5 suggested no discriminative ability. We further identified the optimal eGFR threshold for predicting antibiotic resistance isolates using the Youden index.

In our analysis, a *p*-value of less than 0.05 was considered statistically significant.

## 3. Results

### 3.1. Characteristics of the Study Group and the Prevalence of UTIs

From 1 January to 31 December 2023, 1526 patients were admitted to the DNMB Clinic, and among them, 1282 (84%) adult patients with T1D or T2D had urine cultures collected and analyzed. The number of positive samples for uropathogens, with a colony count higher than 10^5^ CFU/mL of urine, was 241 (18.8%), collected from the same number of diabetic patients who were included in the present study analysis. Thus, the prevalence of UTIs in our population of DM patients was 18.8% (95% CI: 16.5–21.3%), higher in patients with T2D compared to patients with T1D (19.7% vs. 11.2%; *p* = 0.0135), and higher in women than in men (34.8% vs. 11.1%, *p* < 0.0001).

The detailed characteristics of the patients from the study group regarding demographic profile and DM status described by specific analyses and DM complications and comorbidities are presented in Table 1.

### 3.2. Characteristics of Patients with UTIs Compared by Type of DM

The 241 diabetic patients with UTIs included in the study group were classified according to the type of DM they had: 16 (6.6%) patients had T1D (UTI-T1D group), while 225 (93.4%) had T2D (UTI-T2D group).

When comparing patients with UTIs regarding the two types of DM, we found that UTI-T2D patients had a significantly older age (70 years vs. 45.5 years, *p* < 0.0001), a longer duration of DM (13 years vs. 4 years, *p* = 0.0278), a higher waist circumference (101 cm vs. 85 cm, *p* = 0.0004), a higher BMI (30.8 kg/m^2^ vs. 22.5 kg/m^2^, *p* = 0.0001) and lower levels of eGFR (74 mL/min/1.73 m^2^ vs. 106 mL/min/1.73 m^2^, *p* = 0.0018), than UTI-T1D patients. The HbA1c and the random plasma glucose level at admission were significantly higher in UTI-T1D patients’ group (HbA1c 10.7% vs. 8%, *p* = 0.0010, and random plasma glucose level at admission is 426 mg/dL vs. 200 mg/dL, *p* = 0.0037, respectively). The comparison of general characteristics of patients by type of DM is presented in Table 2.

Regarding the presence of DM complications and comorbidities in patients with UTIs, there were statistically significant differences between the two DM groups. Thus, we noticed that among diabetic patients with UTIs, those with T1D had more frequent DKA compared to patients with T2D (68.8% vs. 18.7%, *p* < 0.0001). By contrast, there were more cases of CKD (40.9% vs. 12.5%, *p* = 0.0248), polyneuropathy (95.1% vs. 68.7%, *p* = 0.0001), coronary artery disease (52% vs. 12.5%, *p* = 0.0023), hypertension (92.9% vs. 25%, *p* < 0.0001), dyslipidemia (78.7% vs. 56.3%, *p* = 0.0394), and heart failure (43.1% vs. 12.5%, *p* = 0.0164), respectively, in the UTI-T2D group vs. the UTI-T1D group. (Table 3)

### 3.3. Diversity of Uropathogens Isolated from UTIs

Of the 241 positive urine cultures, 228 (94.6%) bacterial strains and 13 (5.4%) *Candida* spp. strains were isolated. The Gram-negative bacilli (GNB) were predominant (187 strains; 77.6%) followed by the Gram-positive cocci (GPC) (41 strains; 17%).

Among the GNB, *Enterobacterales* were the best represented, with *E. coli* being the most frequent (136 strains; 56.4%), followed by *Klebisella pneumoniae* (31 strains; 12.9%) and *Proteus mirabilis* (8 strains; 3.3%). Among the GPC, *Enterococcus* spp. was predominant (23 strains; 9.5%), followed by *Streptococcus agalactiae* (14 strains; 5.8%).

Studying the features regarding the etiology of UTIs in the two types of DM, we noticed a different behavior between the groups. In UTI-T1D patients, there was a balance in the distribution of GNB (43.8%), GPC (25%), and fungal isolates (31.2%), while in UTI-T2D patients, the GNB were predominant (80%) and the incidence of fungal infection decreased significantly (3.6%). In this regard, there was a significantly higher frequency of GNB (*p* = 0.0008), compensated by a significantly lower number of fungal strains (*p* < 0.0001) in UTI-T2D samples compared to UTI-T1D samples.

The comparative study of the species isolated in the urine samples of the two DM groups showed a highly statistically significant increase in *E. coli* (*p* < 0.0017) and a decrease in *Candida* spp. (*p* < 0.0001) for T2D vs. T1D patients. The other germs showed no statistically significant differences regarding their distribution in the two types of DM (Table 4).

### 3.4. Antimicrobial Resistance (AMR) Pattern

AST interpretation was performed according to EUCAST breakpoint tables version 13.0 [20], and the inclusion of pathogens in the MDR and XDR categories was performed according to the definition of Magiorakos et al. [21].

The study of AMR showed that 55 strains (22.8%) belonged to the MDR phenotype, 28 strains (11.6%) were ESBL producers, and 4 strains (1.6%) were CRO, respectively. Four XDR strains were identified, and all of them were in the UTI-T2D groups’ samples (Table 5).

Among the identified MDR species, GNB were the most numerous, through *E. coli* (56.3%) and *K. pneumoniae* (23.6%), while GPC were represented mainly by *S. aureus* (3.6%) and *Streptococcus* group B (*S. agalactiae*) (3.6%). The ESBL-producing strains belonged to *E. coli* (50%) and *K. pneumoniae* (39.2%), as well as, with very low frequencies, to *P. aeruginosa* and *Citrobacter* spp. The CRO isolates (four strains) belonged to *K. pneumoniae* (two isolates), *P. aeruginosa*, and *Myroides* sp. The four XDR strains were two *K. pneumoniae*, one *E. coli* and one *Myroides* sp., respectively.

Most of the strains with acquired resistance phenotypes were identified in the urine samples of T2D patients (92.7% of the total number of MDR, 92.8% of the total ESBL isolates, and 75% of the total CRO, respectively). Species with acquired AMR in T1D patients’ samples were represented by a single strain of ESBL-producing and CR *K. pneumoniae*, one strain of ESBL-producing and CR *P. aeruginosa*, from the GNB group, and by one strain of MRSA and one strain of MDR *Enterococcus* spp., from the GPC group, respectively. We found no statistically significant differences regarding the distribution of AMR patterns in the two types of DM.

We tested whether the presence of MDR or ESBL resistance patterns is associated with the level of glycemic control (estimated by the HbA1c) or with the eGFR level. We found an inverse association between the eGFR level and the occurrence of MDR and ESBL patterns, respectively (the decrease in the eGFR correlated with the increase in the number of resistant strains, both for MDR (*p* = 0.0489) and ESBL (*p* = 0.0001) patterns). HbA1c level did not associate with the presence of AMR patterns (Table 6).

Multiple logistic regression showed that eGFR was the only statistically significant factor associated with the emergence of MDR and ESBL isolates (OR = 0.98, 95% CI: 0.97–0.99; *p* = 0.042 and OR = 0.97, 95% CI: 0.95–0.98; *p* < 0.001, respectively).

**Figure 1:**
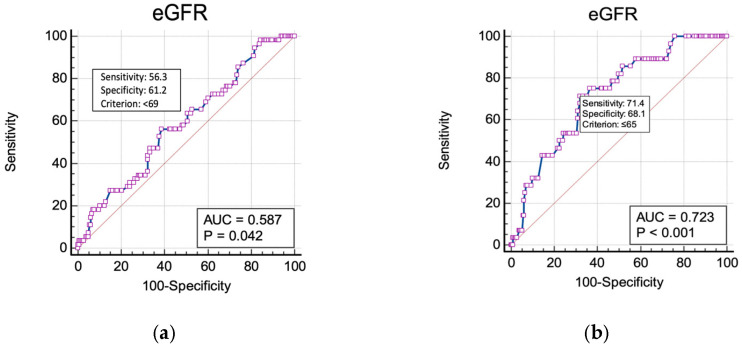
ROC curve analysis of eGFR for (**a**) MDR isolates and for (**b**) ESBL isolates.

The ROC curve analysis of eGFR as a predictor for UTIs with MDR isolates demonstrated a sensitivity of 56% and a specificity of 61% (AUC = 0.587, *p* = 0.0419) at an eGFR threshold <69 mL/min/1.73 m^2^ (Figure 1a). Likewise, the ROC curve analysis of eGFR as a predictor for UTIs with ESBL isolates showed a sensitivity of 71% and a specificity of 68% (AUC = 0.723, *p* < 0.001) at an eGFR threshold <65 mL/min/1.73 m^2^ (Figure 1b).

## 4. Discussion

According to our study, the prevalence of UTIs in patients with DM was 18.8%, which is in line with other studies conducted in Europe (Italy 18.1%, Portugal 16.2%) [22,23]. Prevalence rates vary in different countries of the world, ranging from 8.2% in the USA [24] to 12.5% in China [25] and being as high as 34.9% in Kuwait [26] and 39.3% in Saudi Arabia [27]. These variations might be explained by the diversity of the geographic area, lifestyle, personal hygiene practice, economic status, and by differences in sample size or study timeframe.

The association between UTIs and gender has been well established, both for the general population and for patients with DM [28,29]. UTIs occurred more frequently in women than in men (34.8% vs. 11.1%, *p* < 0.0001) in our population of diabetic patients, consistent with the previous findings in the literature [17,23,25,26]. Differences between genders may be related to the shorter urethra and its anatomical proximity to the anus in women [10], but another hypothesis is that high estrogen levels play an important role in increasing the risk of UTIs in women [29].

Numerous studies have investigated the characteristics of UTIs in patients with T2D, but only a few were conducted in patients with T1D [18]. Therefore, we evaluated the particularities of UTIs in patients with T1D compared to those with T2D.

The present study revealed significant differences in patients with UTIs according to the type of DM, as, for example, in terms of age, the duration of DM, BMI, waist circumference, eGFR level, median HbA1c, and the presence of acute and chronic complications of the disease. In general, patients with T2D are usually older, have a higher BMI, and a higher waist circumference than patients with T1D [2], differences that were also notable in our population of patients with UTIs. Also, the duration of DM was longer in UTI-T2D vs. UTI-T1D patients, meaning the risk of chronic complications of DM, and of CKD in particular, is increased in this category of patients, which may explain the lower eGFR levels observed in this group. Indeed, the presence of CKD was higher in the UTI-T2D group compared to the UTI-T1D group. Moreover, lower eGFR levels also correlated with the emergence of AMR bacterial isolates in our study (MDR- and ESBL-producing strains, respectively).

On the other hand, T1D patients are more prone to developing metabolic complications due to the absolute insulin deficiency that is characteristic of this type of the disease [2]. Acute, life-threatening episodes of DM, like DKA, were significantly more frequent in UTI-T1D than in UTI-T2D patients.

Several previous reports have demonstrated an association between elevated HbA1c levels and the presence of UTIs [26,30,31,32]. However, the HbA1c level did not seem to correlate with the emergence of AMR bacterial isolates in the urine of diabetic patients from our study.

Analysis of the etiology of UTIs in diabetic patients in the present study revealed that *E. coli* was the species with the highest frequency for both DM groups, but with a significantly higher incidence for T2D. It is important to note that in T1D patients, *E. coli* isolates were sensitive to the tested antibiotics and did not cause therapeutic concerns. In T2D patients, about one-fourth of the *E. coli* strains were MDR, 10.53% were ESBL producers, and 31.58% were resistant to sulfonamides. One *E. coli* XDR isolate was also identified in the T2D group, which may result in a long-term course of UTI and limited therapeutic options.

The increased AMR of *E. coli* identified in the UTI-T2D samples can be explained by the prolonged evolution of T2D and the high frequency of CKD recorded in these patients, which implies repeated infection episodes with a progressive evolution, depending on the patient’s adherence to treatment.

*K. pneumoniae* was the second most frequent and clinically significant isolated bacterium. Its incidence was low in both DM groups (almost 12%), but the importance of these isolates is given by the constant presence of the acquired resistance phenotypes. In the UTI-T1D group, two isolates of *K. pneumoniae* were identified, one of them being an MDR and ESBL producer. In the UTI-T2D group, among 29 identified isolates, 41.3% were MDR, 34.4% were ESBL producers, and 6.9% were CRO, respectively; they were also associated with sulfonamide resistance (24.1%). The high-AMR behavior of *K. pneumoniae* was also highlighted by the identification of two colistin-resistant isolates in the UTI-T2D group, which in this context, were classified as XDR.

Our AMR results show that *K. pneumoniae* is a potentially high-resistant isolate, so its identification in samples of DM patients is an indicator of the severity of UTIs. The *K. pneumoniae* isolates with a high-resistance behavior were identified in patients with advanced CKD associating important vascular and neurological complications, with previous hospitalizations and antibiotic treatments, which explains the accumulation of resistance mechanisms.

The present study showed an increase in the AMR trend of GNB, especially in *E. coli* and *K. pneumoniae* (56.3% and 23.6% MDR isolates, respectively), compared to another study conducted in our clinic 7 years ago, which reported MDR strains with ESBL production in only 4.3% of *E. coli* and 10.8% of *K. pneumoniae* isolates, respectively [31].

Furthermore, we want to highlight the situation of another XDR-GNB, the *Myroides* isolate, from the UTI-T2D group, an opportunistic pathogen, which can rarely be responsible for infections, including UTIs, in the immunocompromised host [33,34]. It is resistant to a wide range of antimicrobial agents, including beta-lactams, monobactams, carbapenems, and aminoglycosides, which implies extremely limited therapeutic options [35]. This was also the case with the strain isolated in the present study, which was sensitive only to colistin. Moreover, the identification of this strain has also been reported in our clinic on other occasions [33].

Regarding the etiology of UTIs, our results are consistent with other studies [11,14,17,22,25,26], while some differences in the rate of AMR were noticed. Thus, Flores-Mireles et al. noted that treatment for UTIs is getting more and more challenging since a variety of AMR mechanisms are spreading. Therefore, enterobacteria such as *K. pneumoniae* and *E. coli* are especially concerning since they have both been found to harbor plasmids that encode ESBLs [14]. At the same time, another study [25] reports an incidence of 50.0% for MDR *E. coli* and 41.7% for *K. pneumoniae*, respectively, in diabetic patients, while Confederat et al. mention that DM raised the incidence of UTIs with ESBL-producing *E. coli* and *K. pneumoniae* [11]. On the contrary, the *E. coli* isolates in another Romanian report [17] showed good antibiotic sensitivity, with only 18.4% of the isolates producing ESBLs, while *K. pneumoniae* showed resistance to cephalosporins, quinolones, gentamycin, and combinations of amoxicillin and clavulanic acid and piperacillin and tazobactam. Similarly to our results, in the study conducted by Miftode et al. [36], 60% for MDR *E. coli* and 18% for MDR *Klebsiella* spp., respectively, were reported.

XDR-GNB strains could be included in the difficult-to-treat resistance category [37], i.e., resistant to all first-line antibiotics (carbapenems, extended-spectrum cephalosporins, and fluoroquinolones). The therapeutic options for these isolates are extremely limited, being represented by combined antibiotic therapy with reserve antibiotics such as colistin, new-generation cyclins, cephalosporins with or without inhibitors, or even phage therapy.

The present study has some limitations to be noted. The fact that only diabetic inpatients were included may lead to the lack of extrapolation of the results to the entire diabetic population. The lack of tests to highlight the molecular mechanisms of AMR could also be a limitation of the study. Last but not least, the study was conducted in only one hospital in the same city, so further investigations should be conducted to validate these results in larger populations.

## 5. Conclusions

The present study brings new data regarding the prevalence of UTIs in diabetic patients from Romania. We found an 18.8% prevalence of UTIs in our DM inpatient population. *E. coli*, followed by *K. pneumoniae* and *Enterococcus* spp. were the most common microorganisms involved in UTIs in diabetic patients. Most of the strains with acquired resistance phenotypes were identified in the urine samples of T2D patients. By identifying the spectrum of uropathogens and their AMR pattern in patients with DM and UTIs, this paper may contribute to optimizing antibiotic treatment, reducing the risk of complications and improving UTI management by tailoring therapies based on AMR profiles. This can help decrease the overuse of antibiotics and prevent recurrent UTIs in diabetic patients. Extensive *studies* are needed to follow up on the molecular mechanisms of AMR in uropathogens from diabetic patients in our country.

## Figures and Tables

**Table 1:** Characteristics of the study sample.

Variable	Patients (n = 241)
Gender ^b^	
Female	184 (76.3%)
Male	57 (23.7%)
Geographic area ^b^	
Urban	134 (55.6%)
Rural	107 (44.4%)
Smoking ^b^	73 (30.3%)
Alcohol consumption ^b^	34 (14.1%)
Age ^a^ (years)	69 [65; 75]
Diabetes type ^b^ (years)	
T1D	16 (6.6%)
T2D	225 (93.4%)
Diabetes duration ^a^ (years)	13 [4; 20]
BMI ^a^ (kg/m^2^)	30 [25.6; 34.6]
Waist circumference ^a^ (cm)	101 [90; 114]
Random plasma glucose level at admission ^a^ (mg/dL)	204 [145; 305]
HbA1c ^a^ (%)	8.17 [6.9; 9.1]
BUN ^a^ (mg/dL)	43 [33.7; 58]
Serum creatinine ^a^ (mg/dL)	0.9 [0.7; 1.1]
eGFR ^a^ (mL/min/1.73 m^2^)	75 [51; 95]
uACR ^a^ (mg/g)	25 [14; 76]
Uric acid ^a^ (mg/dL)	5.6 [4.5; 6.9]
Diabetes complications ^b^	
DKA	53 (22%)
Retinopathy	80 (33.2)
CKD	94 (39%)
Polyneuropathy	225 (93.4%)
Coronary artery disease	119 (49.4%)
Cerebrovascular disease	55 (22.8%)
Peripheral artery disease	30 (12.4%)
Comorbidities ^b^	
Hypertension	213 (88.4%)
Dyslipidemia	186 (77.2%)
Heart failure	99 (41.1%)

BMI = body mass index, HbA1c = glycated hemoglobin, BUN = blood urea nitrogen, eGFR = estimated glomerular filtration rate (calculated using CKD-EPI creatinine equation 2021 [19]), uACR = urine albumin–creatinine ratio, DKA = diabetic ketoacidosis, CKD = chronic kidney disease. ^a^ Continuous variables are indicated by their median (interquartile range). ^b^ Categorical variables are represented by the number of individuals and the absolute frequency (percentage) in the sample.

**Table 2:** Comparison of the characteristics of patients with UTIs by type of DM.

Variable ^a^	UTI-T1D Patients (n = 16)		UTI-T2D Patients (n = 225)		*p* *
	Median	Average Rank	Median	Average Rank	
Age (years)	45.5000	43.0000	70.0000	126.5467	*<0.0001*
Duration of DM (years)	4.0000	83.6562	13.0000	123.1317	*0.0278*
HbA1c (%)	10.7000	176.6250	8.0000	117.0444	*0.0010*
Random plasma glucose level at admission (mg/dL)	426.5000	169.8750	200.0000	117.5244	*0.0037*
Waist circumference (cm)	85.0000	61.6250	101.0000	125.2222	*0.0004*
BMI (kg/m^2^)	22.5000	56.7187	30.8000	125.5711	*0.0001*
Serum creatinine (mg/dL)	0.8000	100.5625	0.9000	122.4533	0.2220
BUN (mg/dL)	40.5000	108.6875	44.0000	121.8756	0.4646
Uric acid (mg/dL)	5.9000	116.2813	5.6000	121.3356	0.7793
eGFR (mL/min/1.73 m^2^)	106.5000	173.6875	74.0000	117.2533	*0.0018*
uACR (mg/g)	15.5000	112.1563	26.0000	121.6289	0.5994

BMI = body mass index, HbA1c = glycated hemoglobin, BUN = blood urea nitrogen, eGFR = estimated glomerular filtration rate, uACR = urine albumin–creatinine ratio. ^a^ Continuous variables. * Mann–Whitney U test.

**Table 3:** Diabetes complications and comorbidities in patients with UTIs compared by type of DM.

Variable ^b^	UTI-T1D Patients (n = 16)		UTI-T2D Patients (n = 225)		*p* *
	No	%	No	%	
DKA	11	68.8	42	18.7	<0.0001
Retinopathy	3	18.8	77	34.2	0.2051
CKD	2	12.5	92	40.9	0.0248
Polyneuropathy	11	68.7	214	95.1	0.0001
Coronary artery disease	2	12.5	117	52	0.0023
Cerebrovascular disease	2	12.5	53	23.6	0.3096
Peripheral artery disease	2	12.5	28	12.4	0.9948
Hypertension	4	25	209	92.9	<0.0001
Dyslipidemia	9	56.3	177	78.7	0.0394
Heart failure	2	12.5	97	43.1	0.0164

DKA = diabetic ketoacidosis, CKD = chronic kidney disease. ^b^ Categorical variables. * Chi-squared test.

**Table 4:** Uropathogens isolated from the urine of diabetic patients.

UTI Pathogens	UTI-T1D Patients	UTI-T2D Patients	*p* *
	No (%)	No (%)	
**Gram-negative bacilli**			
*E. coli*	3 (18.8%)	133 (59.1%)	0.0017
*Proteus mirabilis*	1 (6.2%)	7 (3.1%)	0.49
*Klebsiella pneumoniae*	2 (12.5%)	29 (12.9%)	0.96
*Enterobacter* spp.	0 (0%)	3 (1.3%)	0.642
*Citrobacter freundii/farmeri*	0 (0%)	1 (0.4%)	0.789
*Klebsiella oxytoca*	0 (0%)	1 (0.4%)	0.789
*Morganella morgagni*	0 (0%)	2 (0.9%)	0.705
*Serratia marcescens*	0 (0%)	1 (0.4%)	0.789
*Pseudomonas* spp.	1 (6.2%)	2 (0.9%)	0.062
*Myroides* sp.	0 (0%)	1 (0.4%)	0.789
**Gram-positive cocci**			
*Staphylococcus saprophyticus*	0 (0%)	1 (0.4%)	0.789
*Staphylococcus aureus*	1 (6.2%)	2 (0.9%)	0.062
*Enterococcus* spp.	1 (6.2%)	22 (9.8%)	0.643
*Streptococcus agalactiae*	2 (12.5%)	12 (5.3%)	0.237
**Yeasts**			
*Candida* spp.	5 (31.2%)	8 (3.6%)	<0.0001

* Chi-squared test.

**Table 5:** AMR patterns of uropathogens.

AMR Pattern	MDR	XDR	ESBL	CRO	R-SXT	R-AG	R-FQ	R-TE	R-CS	MRSA	MRSCN
No	55	4	28	4	54	11	25	10	1	1	1
%	22.8	1.6	11.6	1.6	22.4	4.5	10.3	4.1	0.4	0.4	0.4

MDR = multidrug-resistant bacteria, XDR = extensively drug-resistant bacteria, ESBL = extended-spectrum beta-lactamases producing Gram-negative bacilli, CRO = carbapenem-resistant organisms, R-SXT = resistance to sulfonamides, R-AG = resistance to aminoglycosides, R-FQ = resistance to fluoroquinolones, R-TE = resistance to cyclins, R-CS = resistance to colistin, MRSA = methicillin-resistant *S. aureus*, MRSCN = methicillin-resistant coagulase-negative staphylococci.

**Table 6:** Association of AMR patterns of uropathogens with different parameters.

Variable	Median	AverageRank	Median	AverageRank	*p* *
	MDR Pathogens (n = 55)		Non-MDR Pathogens (n = 186)		
HbA1c (%)	8.0000	112.3091	8.2000	123.5699	0.2925
eGFR (mL/min/1.73 m^2^)	68.0000	104.7364	77.0000	125.8091	0.0489
	**ESBL pathogens (n =28)**		**Non-ESBL pathogens (n = 213)**		
HbA1c (%)	8.0800	98.4464	8.1700	123.9648	0.0686
eGFR (mL/min/1.73 m^2^)	53.0000	73.4821	79.0000	127.2465	0.0001

MDR = multidrug-resistant bacteria, ESBL = extended-spectrum beta-lactamases producing Gram-negative bacilli, HbA1c = glycated hemoglobin, eGFR = estimated glomerular filtration rate. * Mann–Whitney U test.

## Data Availability

Data are available upon request from the corresponding author.

## Abbreviations

DM	diabetes mellitus;
T1D	type 1 diabetes;
T2D	type 2 diabetes;
UTI	urinary tract infection;
ASB	asymptomatic bacteriuria;
HbA1c	glycated hemoglobin;
DKA	diabetic ketoacidosis;
CKD	chronic kidney disease;
eGFR	estimated glomerular filtration rate;
AST	antimicrobial susceptibility testing;
MIC	minimum inhibitory concentration;
AMR	antimicrobial resistance;
MRSA	methicillin-resistant S. aureus;
ESBLs	extended-spectrum beta-lactamases;
CRO	Carbapenem-resistant organisms;
MDR	multidrug-resistant bacteria;
XDR	extensively drug-resistant bacteria;
OR	odds ratio;
ROCs	receiver-operating characteristics;
AUC	area under the ROC curve;
CI	confidence interval;
BMI	body mass index;
BUN	blood urea nitrogen;
uACR	urine albumin–creatinine ratio;
GNB	Gram-negative bacilli;
GPC	Gram-positive cocci.
